# Urofacial (Ochoa) syndrome with a founder pathogenic variant in the *HPSE2* gene: a case report and mutation origin

**DOI:** 10.1007/s13353-024-00896-7

**Published:** 2024-08-16

**Authors:** Manuela Del Valle-Peréz, Alejandro Mejía-García, Dayana Echeverri-López, Katherine Gallo-Bonilla, Johanna A. Tejada-Moreno, Andrés Villegas‑Lanau, Mateo Chvatal-Medina, Jorge E. Restrepo, Gina Cuartas-Montoya, Wildeman Zapata-Builes

**Affiliations:** 1 Grupo Infettare, Facultad de Medicina, Universidad Cooperativa de Colombia, Medellín, Colombia; 2 Grupo de Genética Molecular (GENMOL), Facultad de Ciencias Exactas y Naturales (FCEN), Universidad de Antioquia, Medellín, Colombia; 3 Grupo Inmunovirología, Facultad de Medicina, Universidad de Antioquia, Medellín, Colombia; 4 Grupo OBSERVATOS, Facultad de Educación Y Ciencias Sociales, Tecnológico de Antioquia – Institución Universitaria, Medellín, Colombia; 5 Facultad de Psicología, Grupo Neurociencia Y Cognición, Universidad Cooperativa de Colombia, Medellín, Colombia

**Keywords:** Urofacial syndrome, HPSE2, LRIG2, Urinary tract, Inverted facial expression, Psychology, Neuropsychology

## Abstract

Urofacial syndrome or Ochoa syndrome (UFS or UFOS) is a rare disease characterized by inverted facial expression and bladder dysfunction that was described for the first time in Colombia. It is an autosomal recessive pathology with mutations in the HPSE2 and LRIG2 genes. However, 16% of patients do not have any mutations associated with the syndrome. Despite the importance of neurobiology in its pathophysiology, there are no neurological, neuropsychological, or psychological studies in these patients. A 30-year-old male from Medellín, Colombia, with a significant perinatal history, was diagnosed with grade 4 hydronephrosis on his first ultrasound test. At 4 months of age, symptoms such as hypomimia, lagophthalmos, and recurrent urinary tract infections started to manifest. Imaging studies revealed urinary tract dilatation, vesicoureteral reflux, and a double collector system on his left side, which led to the diagnosis of UFS. Multiple procedures, including vesicostomy, ureterostomy, and enterocystoplasty, were performed. At 20 years of age, he achieved urinary sphincter control. Genetic analysis revealed a founder pathogenic variant, *c.1516C* > *T (p.Arg506Ter)*, in the HPSE2 gene, which produces a truncated protein that lacks 86 amino acids. This variant is classified as pathogenic according to the ClinVar database for UFS. The mutation age is approximately 260–360 years, and the two alleles share a 7.2–7.4 Mb IBD segment. Moreover, we detected European local ancestry in the IBD segment, which is consistent with a Spanish introduction. Neurological examination, neuropsychological assessment, and psychological testing revealed no abnormalities, except for high stress levels. Clinical analysis of this patient revealed distorted facial expression and detrusor-sphincter dyssynergia, which are typical of patients with UFS. Genetic analysis revealed a pathogenic variant in the HPSE2 gene of European origin and a mutation age of 260–360 years. From a neurological, neuropsychological, and psychological (emotional and personality) perspective, the patient showed no signs or symptoms of clinical interest.

## Introduction

In the early 1960s in Colombia, Dr. Bernardo Ochoa observed a series of patients with urinary incontinence, recurrent urinary tract infections (UTIs), and clinical and radiological findings such as neurogenic bladder. These patients showed an inverted facial expression when they tried to laugh or smile (). In 1979, Dr. Elejalde performed the first genetic evaluation in a case series and coined the term urofacial or Ochoa syndrome (UFS or UFOS) to describe these findings, which are recognized as rare diseases with unknown prevalence (Alqasem et al. 2021). In the last 50 years, more than 150 cases have been reported worldwide, most of which were from Colombia, although others have been reported in Argentina, the United States, Europe, Africa, and Asia (Osorio et al. 2021). A study of 50 patients conducted by Dr. Ochoa between 1965 and 1991 concluded that UFS is usually diagnosed in childhood and shows no difference between sexes (Ochoa 1992).

The UFS is characterized by voiding dysfunction due to dyssynergia between the detrusor muscle and the external striatum sphincter, which leads to an exaggerated increase in vesical pressure during urination. It is accompanied by dilation of the bladder and superior urinary tract without obstruction or neurological disease (Ochoa and Gorlin 1987). Normal urination is caused by a reflex in the reticular formation located in the brainstem, anatomically near the origin of the facial nerves, causing partial paralysis and bilateral facial muscle weakness (Escala et al. 1998).

UFS is an autosomal recessive pathology associated with mutations in the *HPSE2* or *LRIG2* genes, which are located on chromosomes 10q23-34 and 1p13.2, respectively (Tu et al. 2014; Stuart et al. 2013**).** Due to its low prevalence, there is a lack of studies on this topic. The *HPSE2* gene encodes the secreted protein heparanase 2c (HPA2c), which binds heparan sulfate proteoglycans (HSPGs). HPSE2 generates the HPA2c protein, which normally inhibits the activity of HPA1. In the absence of HPA2, HPA1 activity increases, interrupting the activity of growth factors and altering the autonomic function of the bladder. HPA2c is expressed in the developing bladder, which suggests that it plays a key role in the structural maturation of the bladder (Woolf et al. 2014).

UFS can manifest from the prenatal period, when ultrasound can reveal a fetal megabladder, hydroureter, and/or hydronephrosis (Stamatiou and Karakos 2010). Other main manifestations of UFS are frequent urinary infections, incontinence or enuresis, constipation, and voiding problems (Stamatiou and Karakos 2010). Additional manifestations include inverted facial expressions (when sad/crying, their expression indicates no emotion; when smiling, their expression suggests sadness/crying), nocturnal lagophthalmos, constipation, and intestinal obstruction (Stamatiou and Karakos 2010). Radiological findings include upper urinary tract dilatation, residual urine, low capacity, poor compliance of the bladder, vesicoureteral reflux, and renal scarring (Hazzab 2021).

The diagnosis of UFS depends on clinical characteristics (inverted facial expression and bladder dysfunction) and the identification of the mutations described above. At least one of these clinical or genetic characteristics is sufficient for case definition, and it is worth mentioning that inverted facial mimicry is a pathognomonic feature of the disease, although it is not always present (Osorio et al. 2021).

Unfortunately, UFS progresses to acute and chronic renal failure, pyelonephritis, and urological sepsis (Arcila and Flórez 2014). Hence, it is essential to make an early diagnosis to prevent renal damage by performing functional studies of the urinary tract, such as urinalysis, urine culture, urinary tract, renal ultrasound, excretory urography, and urodynamic studies (Arcila and Flórez 2014).

HPA-2 protein alterations occur in some brain regions and may affect neurological, neuropsychological, or psychological functioning (Zhang et al. 2019; García et al. 2017). However, there is a lack of studies on this topic. Therefore, neurological, neuropsychological, and psychological analyses were performed to explore alterations in these behavioral domains that may cause psychosocial maladjustment. The results may improve the mental health and well-being of people with this medical condition.

The objective of UFS treatment is to achieve adequate vesical evacuation and, most importantly, to prophylactically prevent kidney injury through the use of antibiotics, clean intermittent catheterization, bowel management, surgical intervention such as bladder augmentation or urinary diversion, anticholinergics, and intravesical botulinum toxin injection if needed (Hazzab 2021).

Below, we present a case report of Ochoa urofacial syndrome, which involves genetic, neurological, neuropsychological, and psychological characterization.

## Case presentation

A 30-year-old male from Medellin, Colombia, with a significant perinatal history, was diagnosed with grade 4 hydronephrosis on his first ultrasound. At 4 months of age, he started to present symptoms such as hypomimia, lagophthalmos, and recurrent UTIs treated with nitrofurantoin. Urinary and renal ultrasound revealed urinary tract dilation and vesicoureteral reflux.

At 2 years of age, an excretory urography showed a double collector system on the patient’s left side, for which a vesicostomy was unsuccessful because the patient had persistent urinary incontinence and recurrent UTIs. The ultrasound was repeated and reported, in addition to persistent postvoid reflux and bilateral pyelocaliceal ectasias.

Five years later, the patient required a ureterostomy and a Mitrofanoff enterocystoplasty, which consisted of an anastomosis of the left and right ureters; a bladder graft was made using tissue from the intestine, and a duct was created to empty the bladder. Afterwards, a postoperative ultrasound revealed mild renal ectasia with minimal dilation, normal ureterovesical union, and adequate postoperative adaptation.

Despite multiple surgical procedures, he experienced persistent urinary incontinence. Ultrasound revealed that nephropathic reflux was still present, along with minimal dilation of the bilateral cavities without obstruction and postvoid residue. Worryingly, he presented a progressive deterioration of renal function since his creatinine levels increased from 0.5 mg/dL in 1997 to 0.7 mg/dL in 2000 and finally to 1.06 mg/dL in 2006.

Finally, he achieved urinary sphincter control at the age of 20 with micturition therapy, along with a frank decrease in UTI recurrence, although he persisted with facial hypomimia, nocturnal lagophthalmos, and sometimes associated with constipation.

**Fig. 1:**
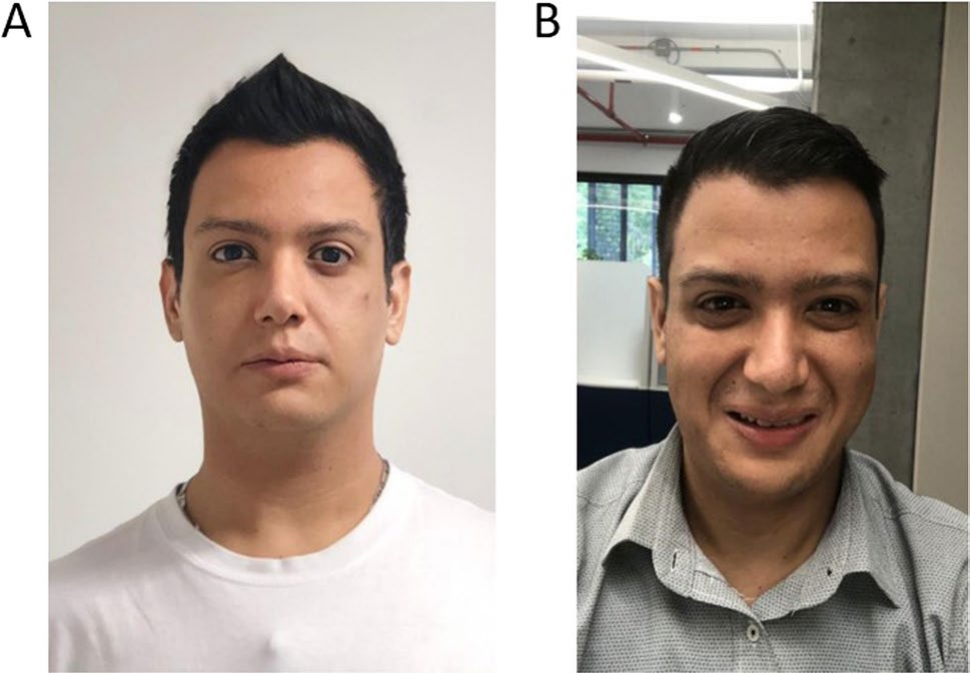
Photography of facial mimicry alterations. Hypomimia (**A**) and inverted facial expression (**B**) when smiling (his expression suggests sadness or crying)

Currently, he has total control of urinary sphincter function with strict schedules of urination every 2 h (Fig. 1).

### *Neuropsychological assessment*

The neurological examination was performed by a clinical neurologist using a standardized clinical evaluation procedure. A neuropsychological assessment was performed using the NEUROPSI, a standardized neuropsychological test to assess memory, attention, and executive functions (Ostrosky-Solís et al. 2003). This test was performed by a clinical neuropsychologist. A clinical psychologist administered three tests to screen for emotional disturbances: anxiety (Beck Anxiety Inventory), depression (Beck Depression Inventory), and stress (Stress Rating Scale) (Kreutzer et al. 2018; Upton 2013; Seara et al. 1996). Additionally, an NEO-FFI personality test was performed (Costa and McCrae 1989).

The neuropsychological findings included, first, no alterations in cranial nerves, as he recognized four out of six odors, had a normal funduscopic examination, showed normal eye movements, had bilateral refractive disorders, had no hearing impairment, was normokinetic, was with normoesthesia and hypomimia, displayed a normal tandem gait, and had muscle tone in all extremities without alterations, mild hyperelastosis with left tricipital, left bicipital osteotendinous reflexes, left brachioradialis, and right brachioradialis hyperreflexia, without pathological reflexes, adequate fine motor skills, and rapid alternating movements without alterations. Regarding the assessment of higher mental functions, he showed anarthria.

**Fig. 2:**
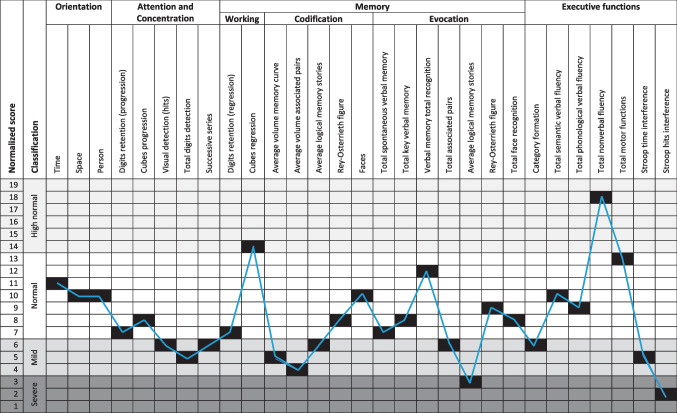
NEUROPSI test results. Tasks and performance on the 29 subtests of the neuropsychological assessment through the NEUROPSI

In his neuropsychological assessment (Fig. 2), mild cognitive impairment in concentration and attention was detected, although working memory was unaffected. The encoding and recall mechanisms of memory had low-normal and mildly impaired functioning. The ability to organize information, concepts, and skills into categories (category formation) was normal. Information processing speed had a normal-high performance. Mild to severe inhibitory control deficits were detected. No anxiety was detected through psychological testing. Low levels of depression were present. The patient showed very high levels of stress. In terms of personality, the patient showed normal-high levels of neuroticism, very low levels of extraversion, normal-low levels of openness to experience, normal-low levels of agreeableness, and normal levels of consciousness.

### Genetic analysis

DNA was extracted from blood samples, and then the exons were captured with the SureSelect V6-Post Capture kit at Macrogen, Inc. The whole exome was sequenced using the HiSeq4000 platform, and the BCL files were converted into FASTQ files with BCL2fastq. Base quality control was performed with FASTQC, and reads were aligned to the hg19 reference sequence from UCSC via the Burrows‒Wheeler Aligner program (bwa 0.7.12) (Bioinformatics et al. 2024; NCBI 2024; Li and Durbin 2009).

The aligned reads were sorted, and the duplicates were marked with Picard-tools 1.130 (Tools et al. 2024). GATKv3.4.0 was used to perform the local indel realigner, base recalibration (for adjusting the Phred quality scores), and variant calling.

A filter step was performed with GATK with quality parameters (McKenna et al. 2010). The VCF file was annotated with SnpEff v4.1 g and wANNOVAR (Cingolani et al. 2012; Wang et al. 2010). The clinical interpretation of the genetic variants was carried out according to the guidelines proposed by the American College of Medical Genetics and Molecular Pathology (Richards et al. 2015). The tools Wintervar and Varsome were used for the classification of candidate variants (Li and Wang 2017; Kopanos et al. 2019).

We found a pathogenic variant, *c.1516C* > *T (p.Arg506Ter)*, in the HPSE2 gene, which is located on chromosome 10 and produces a truncated protein that lacks 86 amino acids (Table 1 and Supplementary Table 1). This variant is classified as a pathogenic variant according to the ClinVar database for UFC. The patient is homozygous (AA) for this variant, and this syndrome is a recessive trait. The A allele has a very low frequency in several populations, with an MAF < 0.001 according to the ExAC and gnomAD databases (Table 1 and Supplementary Table 2). The evolutionary conservation predictors showed that this site is highly conserved (Table 1 and Supplementary Table 3). Additionally, several pathogenicity predictors, such as Fathm, MKL, and Mutation Taster, showed deleterious effects. Other predictors showed values close to a deleterious effect, such as CADD and DANN scores, while others failed to evaluate pathogenicity, such as SIFT and Polyphen2 (Table 1 and Supplementary Table 4).

**Table 1:** Description of candidate variants under the prioritization criteria identified with the ANNOVAR tool in the D family

Chr	Position	Ref	Alt	Gene	AA change	dbSNP	1000G	ExAC	gnomAD	GenoCanyon	fitCons	GERP + + RS	SIFT	Polyphen2HDIV	Polyphen2HVAR	CADD	Genotype
10	100,242,490	G	A	HPSE2	NM_001166246.1:c.1516C > T (p.Arg506Ter)	rs267606866	-	0.00002471	0.00001591	1	0.601	3.41	**-**	**-**	**-**	**13.811**	AA

**Notes and abbreviations:** *Chr* chromosome, *Start* variant start position, *Position* position in the hg19/CHR37 reference genome, *Ref* reference allele, *Alt* alternate allele, *Gene* gene name, *AA change* amino acid change, *dbSNP* variant identifier in the dbSNP database, *1000G* allele frequency in the 1000 Genomes Database (all populations), *ExACFreq* allele frequency in the ExAC 65000 database (all populations), *gnomAD* allele frequency in gnomAD database, exome data (all populations), *GenoCanyon* conservation scores calculated with the GenoCanyon tool (conserved region = scores ~ 1), *fitCons* conservation scores calculated with the fitCons tool (conserved region =  ~ 1), *GERP* +  + *RS* conservation scores obtained with the GERP +  + RS tool (conservation region = scores > 4.4), *SIFT* pathogenicity prediction with the SIFT tool (*D* deleterious, *T* tolerant), *Polyphen2HDIV* pathogenicity prediction with the PolyPhem2 tool for Mendelian disease variants (*D* damaging, *P* possibly damaging, *B* benign, *U* unknown), *Polyphen2HVAR* pathogenicity prediction with the PolyPhem2 tool for all human disease-causing mutations (*D* damaging, *P* possibly damaging, *B* benign, *U* unknown)

### Variant detection

According to the results of the Varsome and Intervar platforms, this variant is classified as pathogenic because of its low frequency in the population, because of clinical evidence, and because multiple lines of computational evidence, such as DANN, FATHMM-MKL, and Mutation Taster, support a deleterious effect on the gene or gene product (Table 2).

**Table 2:** Clinical interpretation of the candidate variants identified in the exome analysis of a patient with UFS

Chr	Gene	Change	dbSNP	Genotype	Varsome	Intervar	Clinvar
10	HPSE2	p.Arg506Ter	rs267606866	AA	PSV1, PM2, PP3, PP5 (pathogenic)	PSV1, PM2, PP3, PP5 (pathogenic)	Pathogenic

**Notes and abbreviations:** *Chr* chromosome, *Gene* name of the gene, *Change* nucleotide/amino acid change, *dbSNP* identifier of the variant in the dbSNP database, *Varsome* classification of variants according to the Varsome platform, *Intervar* classification of the variants according to the Intervar platform, *PSV1* null variant (nonsense, frameshift, canonical +—2 splice sites, initiation codon, single or multiexon deletion) in a gene in which LOF is a known mechanism of disease, *PM2* absent from controls (or at extremely low frequency if recessive) in the Exome Sequencing Project, 1000 Genomes Project, or Exome Aggregation Consortium, *PP3* multiple lines of computational evidence support a deleterious effect on the gene or gene product (conservation, evolutionary, splicing impact, etc.), *PP5* reputable sources have recently reported variants as pathogenic, but the evidence is not available to the laboratory to perform an independent evaluation

### Mutation age estimation

The filtered VCF file from the patient was processed using Plink2 (www.cog-genomics.org/plink/2.0/) to include only variants from chromosome 10, leading to a total of 4700 variants. Then, we downloaded 1000 genomes from the PLINK2 resource website, and we selected individuals from Medellin (CLM, 94 individuals) and European individuals from Utah (CEU, 99 individuals). We merged this 1000 Genomes subset dataset with the VCF from the patient using PLINK1.9 (https://www.cog-genomics.org/plink/) and the flag-geno 0.004 to include SNPs that are present in both the patient and the 1000 Genomes Project individuals, resulting in a final VCF with 4106 variants.

To increase the SNP density on chromosome 10, we performed genotype imputation using the TOPMED imputation server with default parameters (Taliun et al. 2021; Das et al. 2016; Fuchsberger et al. 2015). After imputation, we obtained a VCF file with 523,270 variants. We preprocessed the VCF file using plink2 with –max-alleles 2 and –snps-only flags to retain the biallelic SNPs. We used a genetic map file in plink format and GRch38 coordinates to estimate recombination rates for reconstructing the ancestral recombination graph (ARG) using ARG-Needle software (Zhang et al. 2023). A final VCF file with 232,825 biallelic SNPs was input into ARG-Needle software (Zhang et al. 2023) to reconstruct the ARG of our patient and the CLM and CEU populations using default parameters and a CLM demography model obtained by ASMC software (https://github.com/PalamaraLab/ASMC_data/tree/main/demographies). The ARG stores the transmission history of all variants present in the sample based on haplotype sharing, allowing us to estimate the age of founder mutations (mutations with a common ancestor). We analyzed the ARG with tskit (https://tskit.dev/tskit/docs/stable/python-api.html) to visualize the ARG at position 98,482,733, and we computed the mutation age using the TMRCA tskit function. However, to ensure that genotype imputation did not alter the TMRCA estimation computed by the ARG-Needle, we reconstructed the ARG using the original variants detected by exome sequencing. For this purpose, 4106 variants were phased using the TOPMED imputation server, and after quality control and removal of variants with small genetic distances, we obtained a VCF file with 2754 variants.

Using the imputed data, we obtained an estimated mutation age of 8.7 generations using the imputed data, corresponding to 261 years ago. Using the original variants detected by exome sequencing, the estimated mutation age was 12.05 generations (361 years ago). In addition, individuals within the CLM population did not coalesce as fast as those within these two haplotypes, consistent with a recent founder mutation. Moreover, we used the IBD_segments function in tskit to identify the length of the identity by descent (IBD) segment that these two haplotypes share, and we found an IBD segment of 7.4 Mb (from 92,195,797 pb to 99,670,601.5 pb) with genotype imputed data and 7.2 Mb (92,330,888 to 99,549,496.5 pb) with exome data.

### Analysis of local ancestry

To identify the continental ancestral origin of the mutation, we used the imputed genotype data to perform local ancestry analysis with RfMix2 (Maples et al. 2013). To construct a reference panel, we downloaded 61 Native American genomes from 5 populations present in the Human Genome Diversity Project (HGDP) from the Plink2 website (Bergström et al. 2020) (https://www.cog-genomics.org/plink/2.0/resources), and we merged them with the Iberian population in Spain (IBS, European ancestry) and the Yoruba (YRI, African ancestry) 1000-genome population (61 individuals from each population to avoid overrepresentation in the reference panel) using BCFtools (Danecek et al. 2021). Briefly, we followed the quality control steps described in the STAR protocol (Carrot-Zhang et al. 2021), and we used RfMix 2.0 (https://github.com/slowkoni/rfmix) to estimate the ancestry along chromosome 10 using default parameters and the genotype imputed VCF file. Global ancestry analysis of chromosome 10 by RfMix revealed that the patient had 81% European ancestry, 16% Native American ancestry, and 3% African ancestry, consistent with previous reports for individuals from Medellin (Rishishwar et al. 2015). As control samples for the local ancestry analysis, we included CLM and CEU individuals, and the results showed high levels of European ancestry for CLM and CEU, as expected, confirming the utility of these sets of markers for ancestry estimation (Supplementary Table 5).

**Fig. 3:**
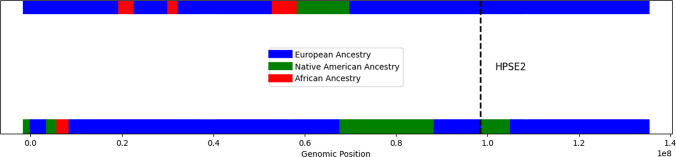
Local ancestry analysis with Rfmix2. Each horizontal block represents a copy of chromosome 10 phased and imputed by TOPMED, showing the continental origin of each haplotype across the chromosome. European ancestry represents the IBS 1000 genomes population (blue), Native American ancestry represents the populations from HGDP (green, see methods for details), and African ancestry represents the YRI 1000 genomes population (red). The vertical dotted line indicates the location of the HPSE2 gene, showing that both gene copies are in European segments. A recombination event with a Native American segment is shown for the second copy close to the HPSE2 gene

We identified that the whole IBD segment that contains the HPSE2 mutation has European ancestry, consistent with a European origin (Fig. 3). We also found a change from European ancestry to Native American ancestry in one of the two haplotypes at 100,332,761, consistent with the break in the IBD segment identified by the ARG-Needle.

## Discussion

UFS is considered a rare disease, and its prevalence is currently unknown worldwide. However, more than 150 cases have been reported in Colombia, which stresses the importance of thoroughly recognizing and characterizing all aspects of this disease (Osorio et al. 2021; Ochoa 1992; Woolf et al. 2019). For proper identification and characterization of this condition, it is crucial to understand the temporal characteristics of clinical manifestations since it is mainly diagnosed in childhood or even from sonographic findings in the prenatal period, such as this case, whose first ultrasound showed significant hydronephrosis.

In the case presented herein, most functional tests and imaging studies were available at an early age, and the patient presented with many of the classical findings of UFS. As noted, the clinical manifestations of this patient will be discussed in two main sections: bladder dysfunction and inverted facial expressions. On the one hand, bladder dysfunctions were widely documented in our patient’s clinical records. The patient debuted with grade 4 hydronephrosis evident on prenatal ultrasound, which is consistent with what has been reported in the literature since the most common UFS findings on fetal ultrasound are megabladder, hydroureter, and hydronephrosis, although these findings are insufficient to make a diagnosis (Osorio et al. 2021; Woolf et al. 2014). During early childhood, he presented one of the usual urinary symptoms described in the early phases of UFS, such as recurrent UTIs. Although, in most cases, recurrent UTIs are an isolated factor and do not necessarily indicate a subjacent disease, our patient subsequently developed a wide variety of symptoms and findings that are characteristic of neurogenic bladder, including urinary incontinence, vesicoureteral reflux, and recurrent UTIs, among others (Ochoa and Gorlin 1987; Rondon et al. 2015). Overall, he presented with the classic urinary bladder dysfunction seen in UFS, described as a nonneurogenic neurogenic voiding dysfunction, where there is detrusor overactivity and sphincter dyssynergia (Ochoa 1992). It is worth noting that in this case, the patient required multiple surgical procedures, and at one point, even his kidney function was impaired, which stresses the degree of bladder dysfunction.

On the other hand, abnormal facial expressions and inversion of facial mimicry were distinguishable in this patient from very early on. Hypomimia was reported in the clinical records and is still evident in the images in Fig. 1. In addition to urinary dysfunction, facial characteristics are crucial to diagnosing UFS (or even suspecting and recognizing it in the first place). Interestingly, in the second half of the twenty-first century, an important number of reports pointed to an association between distortions in facial characteristics and renal impairment (Wang et al. 1999; Teebi and Hassoon 1991; Potter 1946). Nevertheless, in the UFS, there is no difference in facial characteristics but rather a dysfunction in expression since their mimicry in a resting state is indistinguishable from that of any other individual. Only upon their smiles does a grimace appear as if they were sad (Osorio et al. 2021; Tu et al. 2014). This is the case for our patient, whose grimace can be seen in Fig. 1 upon being instructed to smile. Hence, in the absence of structural deficits and only explained by a functional alteration, the finding of inverted facial mimicry corresponds to the pathognomonic finding of UFS, which was indeed diagnosed by competent clinical staff very early in his life and ratified upon clinical examination by our research personnel.

Interestingly, our patient presented nocturnal lagophthalmos, an additional clinical finding in UFS. Although the general pathophysiology of this clinical manifestation is unknown, it might be related to the exact dyssynergia mechanism involved in detrusor-sphincter incoordination between the facial frontalis muscle and the orbicularis oculi muscle (Tu et al. 2014). Even more important than the clinical characteristics, which are unequivocally related to UFS, are the imaging reports that showed abnormalities in the excretory system and apparent vesicoureteral reflux, among others. It must be stressed that further complications, including dilation of cavities and pyelonephritis, along with the progressive impairment of kidney function, put him at risk of suffering future kidney complications related to chronic kidney disease. This evidence, in addition to genetic confirmation through the identification of the pathogenic *HPSE2* gene variant, was the final jewel in the crown of this undisputable diagnosis and will be discussed next.

Regarding the genetic classification, which might be one of the most vital points of our report, we identified a pathogenic variant in the *HPSE2* gene in a patient with UFS. This variant has been previously reported in individuals from Colombia with this syndrome (Pang et al. 2010). In this country, Dr. Ochoa has found a high proportion of patients with this syndrome in Antioquia state, with reports of 17 families with UFS syndrome, where at least one-third of the families lived in four towns located northeast of Medellín (Ochoa and Gorlin 1987; Ochoa 2004). A study in 2010 identified the *c.1516C* > *T (p.Arg506Ter)* mutation in 31 UFS patients from 119 families in Colombia (Wang et al. 1999; Pang et al. 2010). This variant is in exon 11, producing a truncated protein that lacks 86 amino acids at the C-terminus of the Hpa2 protein (Pang et al. 2010). It was found in several patients diagnosed by Dr. Ochoa, and they shared the same haplotype in a region that included the *HPSE2* gene, consistent with a founder effect (Woolf et al. 2014; Pang et al. 2010**).** However, the report did not include the length of the haplotype shared, and they did not estimate mutation age or origin, which could be critical for personalized medicine and for identifying regions with a higher frequency of this variant across Colombia (Pang et al. 2010). Consequently, we used the length of the haplotype to estimate the mutation age, finding an origin of approximately 260–360 years, consistent with the history of Colombia and the foundation of Medellin. In addition, we identified a segment of 7.2–7.4 Mb, demonstrating that these two alleles carried by the proband were inherited from a common ancestor (as shown in the inferred ARG). Due to the high European ancestry and the history of founder mutations introduced by Spanish individuals (Lalli et al. 2014), we provided evidence that this mutation was introduced by a Spanish individual into the Medellin population. Moreover, both exome and imputed data showed similar mutation ages and IBD lengths, and local ancestry analysis was able to identify the end of the IBD segment broken by recombination with a native American segment, showing consistency across methods to identify the segment that contains the HPSE2 mutation.

The *HPSE2* gene encodes three mRNAs; the longest form generates a secreted protein called HPA2c, which binds to heparan sulfate proteoglycans (HSPGs). Among the various functions of HPA-2, there are two upon which particular emphasis must be placed on UFS. First, regarding HPA-2 in the urinary tract, the HPA-2 protein is present in the nerve trunks that invade the human fetal bladder, and some HPA-2-positive neurons are in contact with the detrusor smooth muscle fibers, demonstrating the presence of the protein in the bladder and its importance in the maturation of the lower renal tract and in this syndrome (Woolf et al. 2014; Daly et al. 2010). While HPA-1 elicits neurogenesis in cell culture, HPA-2 inhibits its activity by binding to HSPGs; thus, the *HPSE2* gene product may modify HPA-1 activity in the ganglia (Woolf et al. 2014; Stuart et al. 2015). In the absence of HPA-2, autonomic nerve function is perturbed, and the cyclic low-pressure storage of urine and complete bladder emptying are impaired (Stuart et al. 2015). The disruption of growth factor activity may be caused by the presence of extracellular matrix molecules with heparan sulfate chains, and the lack of HPA-2 results in an imbalance in the regulation of these molecules by heparan sulfate (Daly et al. 2010). Second, regarding the role of HPA-2 in facial mimicry, RT‒PCR assays revealed high expression of *HPSE2* in the facial muscle, which is consistent with the distorted facial expression of patients with UFS, although much of this phenomenon has been explored (Pang et al. 2010).

The nonsense mutation found in the patient (*c.1516C* > *T, p.Arg506Ter*) led to a premature stop codon in the transcript, and the protein is expected to lack 86 amino acids at the C-terminus. Although the protein has no domain at this site (506–592), there is an asparagine at position 543 that is conserved in humans, mice, and Xenopus, and it is a critical amino acid for heparan sulfate binding (Roberts et al. 2014). In addition to our findings, we encountered a report of 3 patients with UFS from a family in Pakistan with a missense mutation at this position (c.1628A > T, p.N543I), confirming that this site is essential for the function of the protein (Mahmood et al. 2012). However, other nonsense mutations have been identified in UFS patients, and they are likely to lead to nonsense-mediated decay of the transcript and therefore an absent protein (Wang et al. 1999). This pathway selectively degrades mRNAs harboring premature termination codons and prevents the production of truncated proteins (Hug et al. 2016). Thus, this could be the pathogenic mechanism of the variant found in our patient, but it should be validated in vitro*.*

Clinical neurological examination did not reveal any abnormalities in nerve function. Neuropsychological impairments in attention, concentration, memory, and executive functioning must be confirmed with a second assessment. Anxiety and depression levels are not of clinical interest. High stress may be due to family, work, or social problems that were not considered in the evaluation, so they might not be related to the UFS per se. The personality profile does not show significant deviations from normality. From a neurological, neuropsychological, and psychological (emotional and personality) perspective, the patient has no signs of clinical interest or symptoms, although due to the absence of studies on these variables, comparing these results is impossible.

Overall, this report describes a patient with classical features of UFS whose diagnosis was confirmed with imaging techniques and whose pathology was successfully managed. From a genetic perspective, we identified a nonsense founder mutation that is partially responsible for disease presentation and seems to play a role in other syndromes involving bladder dysfunction. From a neuropsychological standpoint, this patient seems to have no symptoms or signs of interest. This report has strengths and limitations. First, due to the ample interdisciplinary team, we carried out not only a clinical characterization of the patient but also a thorough neuropsychological assessment and a genetic analysis. Additionally, we could rely on the clinical records, which provide detailed information before birth, allowing us to analyze the data better considering the entire patient history. However, we must also comment on limitations, and the main weakness is the relative lack of comparability of our data, since there are no other reports regarding neuropsychological assessments of these patients, so further research on this matter is warranted.

## Data Availability

The data presented in this study are available upon reasonable request from the corresponding author.
